# Overcoming imatinib resistance conferred by the *BIM* deletion polymorphism in chronic myeloid leukemia with splice-switching antisense oligonucleotides

**DOI:** 10.18632/oncotarget.20658

**Published:** 2017-09-06

**Authors:** Jun Liu, Malini Bhadra, Joanna Rajeswary Sinnakannu, Wan Lin Yue, Cheryl Weiqi Tan, Frank Rigo, S.Tiong Ong, Xavier Roca

**Affiliations:** ^1^ School of Biological Sciences, Nanyang Technological University, Singapore; ^2^ Cancer and Stem Cell Biology Signature Research Programme, Duke-NUS Medical School, Singapore; ^3^ CN Yang Scholars Programme, Nanyang Technological University, Singapore; ^4^ Ionis Pharmaceuticals, Carlsbad, California, USA; ^5^ Department of Haematology, Singapore General Hospital, Singapore; ^6^ Department of Medical Oncology, National Cancer Centre Singapore, Singapore; ^7^ Department of Medicine, Duke University Medical Center, Durham, North Carolina, USA

**Keywords:** alternative splicing, *BIM*, chronic myeloid leukemia, imatinib, antisense oligonucleotides

## Abstract

Many tyrosine kinase-driven cancers, including chronic myeloid leukemia (CML), are characterized by high response rates to specific tyrosine kinase inhibitors (TKIs) like imatinib. In East Asians, primary imatinib resistance is caused by a deletion polymorphism in Intron 2 of the *BIM* gene, whose product is required for TKI-induced apoptosis. The deletion biases *BIM* splicing from exon 4 to exon 3, generating splice isoforms lacking the exon 4-encoded pro-apoptotic BH3 domain, which impairs the ability of TKIs to induce apoptosis. We sought to identify splice-switching antisense oligonucleotides (ASOs) that block exon 3 but enhance exon 4 splicing, and thereby resensitize *BIM* deletion-containing cancers to imatinib. First, we mapped multiple *cis*-acting splicing elements around *BIM* exon 3 by minigene mutations, and found an exonic splicing enhancer acting via SRSF1. Second, by a systematic ASO walk, we isolated ASOs that corrected the aberrant *BIM* splicing. Eight of 67 ASOs increased exon 4 levels in *BIM* deletion-containing cells, and restored imatinib-induced apoptosis and TKI sensitivity. This proof-of-principle study proves that resistant CML cells by *BIM* deletion polymorphism can be resensitized to imatinib via splice-switching *BIM* ASOs. Future optimizations might yield a therapeutic ASO as precision-medicine adjuvant treatment for *BIM*-polymorphism-associated TKI-resistant CML and other cancers.

## INTRODUCTION

Small-molecule targeting of mutant oncoproteins in human cancers has resulted in significant improvements in progression free survival (PFS) and overall survival (OS) compared to conventional chemotherapy [1–3]. In particular, inhibition of BCR-ABL1 by the tyrosine kinase inhibitor (TKI) imatinib (also known as Gleevec or Glivec) in patients with chronic myeloid leukemia (CML) has transformed a previously deadly disease into a chronic illness [4]. Similarly, other TKIs like erlotinib exhibit >70% response rates in non-small cell lung cancer (NSCLC) patients with epidermal growth factor receptor (EGFR) activating mutations [5–7]. Other human cancers like c-KIT-driven gastrointestinal stromal tumours and BRAF-driven melanomas also have available TKIs. The BIM (BCL2-Interacting Mediator of cell death, also known as BCL2L11) protein is a BH3-only proapoptotic member of the BCL-2 family that is absolutely required for the cancer killing of such drugs via the intracellular or mitochondrial pathway, elicited via upregulation of its expression at different levels [8–14]. However, despite the high overall TKI response rates, significant heterogeneity in both the depth and duration of responses exists [15–17].

We previously discovered a common 2,903-base pair (bp) deletion polymorphism in the *BIM* gene that contributed to response heterogeneity in patients with CML and epidermal growth factor receptor-mutated non-small cell lung cancer (EGFR-NSCLC). The deletion allele is present in East Asians and Latin Americans with carrier frequency 13-16%, and absent in Caucasians and Africans [18, 19]. While the presence of the *BIM* deletion reduces the first-line response to imatinib in CML patients [19], in EGFR-NSCLC patients it predicts an inferior OS compared to individuals without the deletion (28.8 vs 40.2 months respectively, p<0.017) [20]. Four independent groups from Taiwan, China, and Japan have replicated our findings [21–25], although two South Korean centres did not show any differences [26, 27], which may possibly be due to genetic differences between East Asians [28, 29].

**Figure 1:**
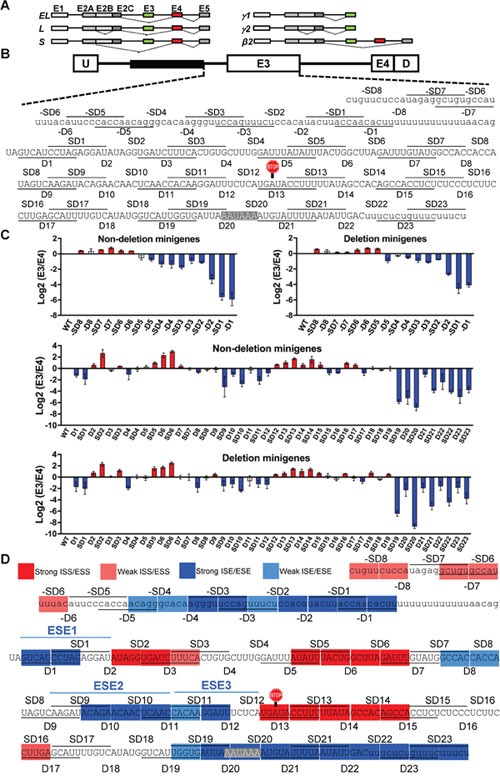
Deletion scan of *BIM* E3 and upstream intronic region reveals many splicing enhancers and silencers **(A)** Schematic of the major proapoptotic (left) and non-apoptotic (right) BIM isoforms. Exons are indicated on top of boxes, introns are depicted as lines, and splicing patterns as thin dashed lines. BIMβ2 is a minor nonapoptotic isoform encoded by an mRNA lacking both E3 and E4. **(B)** Design of the *BIM* minigene with boxes as exons and lines as introns. U and D are adenoviral exons, and the 2,903-bp polymorphic fragment is shown as black box. The 10-nt deletions and staggered deletions (D and SD series) are consecutively indicated as lines and spaces within the sequences. The E3 stop codon (UGA) and polyadenylation signal (AAUAAA) are highlighted. **(C)** Log2(E3/E4) ratios by real-time RT-PCR for the D and SD series in Δ10 (nondeletion minigenes) or Δ11 (deletion minigenes), with red and blue indicating silencers and enhancers, respectively, with a P<0.05 compared to WT minigene using Student's t-test. **(D)** Color-coded map of ESEs, ISEs, ESSs and ISSs within and upstream E3. Strong and weak are respectively defined by ≥50 or ≥30% splicing change by the deletion compared to wild-type (see MATERIALS AND METHODS).

BIM expression is largely regulated by alternative splicing, which generates three major proapoptotic isoforms named BIMEL (extralarge), BIML (large) and BIMS (small), and two isoforms that are not proapoptotic collectively named as BIMγ (with BIMγ1 and BIMγ2) (Figure 1A). BIMEL, BIML and BIMS mRNAs all contain exon 4 (E4) while BIMγ isoforms include exon 3 (E3) instead [19, 30]. Mechanistically, the deletion polymorphism biases alternative splicing away from E4 toward E3, resulting in decreased expression of E4-containing isoforms, and increased E3/E4 ratio [19]. E3 and E4 cannot be included in the same spliced transcript because E3 lacks a 5’ splice sites (5’ss) to be connected with the 3’ splice sites (3’ss) of E4, but instead E3 is a terminal exon with its own canonical polyadenylation signal. Because only E4 encodes the pro-death BH3 domain of BIM, the patients with the deletion exhibit impaired ability to upregulate BH3-containing BIM protein isoforms by TKIs, thus resulting in intrinsic TKI resistance. We also found that the 2,903-bp polymorphic fragment contains multiple and redundant Intronic Splicing Silencers (ISSs), and that the last 322-nucleotide (nt) of this segment is sufficient to recapitulate the repressive effects on E3 by the whole fragment, with an important 23-nt ISS at its 3’ end [31]. Previous studies also revealed that the *trans*-acting factor serine/arginine-rich splicing factor (SRSF1) promotes E3 inclusion [32], and that Polypyrimidine Tract Binding Protein 1 and hnRNP C repress it [31]. However, the binding sites of these factors in the *BIM* transcript remain to be identified, as well as additional activators and repressors of E3 inclusion. Furthermore, SRSF2 and SRSF6 were also shown to increase *BIMS*, which encodes the most potent of all proapoptotic splice isoforms [33, 34].

Beyond the splicing effects of the *BIM* deletion, alternative splicing is a commonly altered mechanism that can fuel tumorigenesis [35], and is also becoming a therapeutic target. Alternative splicing connects exons in different ways to generate different mRNAs from one primary transcript [36], and largely accounts for the complexity of the transcriptome and proteome in humans [37]. Each alternative splicing event is usually regulated by many *cis*-acting elements and their cognate *trans*-acting factors, as well as by RNA structure, transcription and chromatin. The essential *cis*-acting splicing elements include the 5’ss and 3’ss, as well as the branch point sequence (BPS) around the lariat adenosine [38]. In addition, exonic or intronic splicing enhancers (ESEs, ISEs) or silencers (ISEs, ISSs) either activate or repress splicing via binding to activators or repressors, such as serine/arginine-rich (SR) proteins and heterogeneous nuclear ribonucleoproteins (hnRNPs) [39]. Splicing is catalyzed by the spliceosome, which is a large and dynamic macromolecule composed of five small nuclear ribonucleoproteins as well as individual polypeptides which assemble on the pre-mRNA in a stepwise manner [40]. A recent structure of yeast catalytic spliceosome [41, 42] has provided significant insights into basic splicing mechanisms but less so into regulation, because the alternative splicing patterns are usually established prior to formation of this complex. Furthermore, as the alternative splicing prediction tools are still inaccurate, elucidation of the regulatory mechanisms for individual splicing events still needs focused studies as the one presented here.

Given the importance of physiologic splicing in regulating growth, survival, and differentiation [36] it is therefore unsurprising that both mutations at *cis*-acting elements as well as mutations or expression alterations of *trans*-acting splicing factors contribute to cancer [35], such as the case of SRSF1 as a potent oncogene in certain tumors [32, 43, 44]. The discovery of frequent mutations in splicing factors associated to myelodysplastic syndromes (MDS) [45] illustrates that splicing plays an important role in myeloid cells, and that their defects can result in pre-malignant syndromes. The most frequently mutated splicing factor in MDS, splicing factor 3 subunit B1, is also the target of antitumor drugs such as spliceostatin A [46]. In addition to somatic mutations, a few known germline polymorphisms can change the splicing patterns and modify the protein's function [47, 48]. Important technological advances have been recently made in the field of splice-switching antisense-oligonucleotides (ASO), which bind and block *cis*-acting splicing elements to change splicing and reconstitute the protein levels [49]. Remarkably, among the various splice-switching ASOs that reached clinical trials, the nusinersen ASO to treat spinal muscular atrophy was approved for clinical use by the US Food and Drug Administration in December 2016, strongly demonstrating that ASOs are real therapeutic drugs [50]. ASOs are also being tested for cancer targets such as the apoptotic factor B-cell Lymphoma X [51, 52], pyruvate kinase M [53] and the signal transducer and activator of transcription 3 beta [54].

In this study we aimed to develop a therapeutic agent that could act directly and specifically (in a DNA sequence-specific manner) to correct the splice-switching defect produced by the *BIM* deletion. The sequence-specificity of therapeutic ASOs may minimize off-target effects, and avoid toxicities associated by other agents reported to overcome *BIM* deletion-mediated TKI resistance, such as BH3 mimetics and HDAC inhibitors, but which suffer from clinically significant side-effects [19, 55–58]. Here, our approach was to first identify the *cis*-acting sequences that regulate *BIM* splicing within and upstream E3, and guided by this information, to design and test novel splice-switching ASOs to directly correct *BIM* splicing and restore TKI sensitivity. We found that as many as eight ASOs effectively redirected *BIM* splicing from E3 to E4, and reconstitute the TKI-mediated responses in two different CML cell lines. Overall, this work shows that it is possible to manipulate *BIM* alternative splicing to re-sensitize cancers to TKIs.

## RESULTS

### Identification of *cis*-acting elements regulating *BIM* E3 alternative splicing

We first systematically identified the *cis*-acting splicing elements in the *BIM* E3 and upstream intronic region that is common among alleles with or without the 2,903-bp deletion polymorphism. We used the Δ10 and Δ11 *BIM* minigenes [31], which have E3 and E4 with flanking shortened intronic regions fused to adenoviral U and D exonic sequences (Figure 1B). Δ10 splices like the full-length *BIM* substrate as it includes the last 322 of the 2,903 bp that are sufficient for the repressive effects of this region, while Δ11 has this fragment removed to recapitulate the splicing patterns of the 2,903-bp deletion allele. Both Δ10 and Δ11 minigenes were used to confirm the effects of deletions and also to identify elements that are specific to the deletion allele, if any. Similar to a previous study [59], in the D (“deletion”) series of constructs, we introduced consecutive deletions of 10 nucleotide (nt) from the third nucleotide of E3 (first nucleotide outside the conserved region of the 3’ss) to the end of this exon. In the SD “staggered deletions” series, the deletions started from the middle of a D deletion to the middle of the next D. The use of D and SD series reduces the possibility that the changes in E3 splicing conferred by each deletion are due to creation of new junctions rather than to removal of a *cis*-acting element. We identified several regions with consecutive and/or overlapping deletions which consistently increased or decreased E3/E4 ratio as measured by real-time RT-PCR using junction reverse primers with proven specificity (Supplementary Figure 1A) which also captured the splicing change conferred by the deletion polymorphism (Supplementary Figure 1B). The changes in E3/E4 ratio due to deletions suggest the presence of ESSs or ESEs which covered the vast majority of E3 sequence (Figure 1C, 1D). Encouragingly, the changes in E3/E4 ratios for all deletions in Δ10 versus Δ11 were almost perfectly correlated (R^2^ = 0.96), proving the reproducibility of this assay. All deletions covering the consensus poly(A) signal and flanking sequences decreased E3 splicing, which agrees with the notion that 3’-end formation and 3’ss recognition of terminal exons enhance each other [60–62]. We also performed a deletion scan of the Intron 2 region between the 2,903-bp polymorphic fragment and the polypyrimidine tract upstream of E3 (negative deletions, –D and –SD). As expected, deletions of the predicted BPS within –SD1 and –D1 strongly reduced E3/E4 ratio, yet deletion of upstream sequences had a milder effect, as these segments might contribute to U2 small nuclear ribonucleoprotein (snRNP) binding to the BPS. In summary, here we unveiled many potential enhancers and silencers that regulate E3 inclusion, some strong and some weak (Figure 1D), and with functional implications for TKI responses.

**Figure 2:**
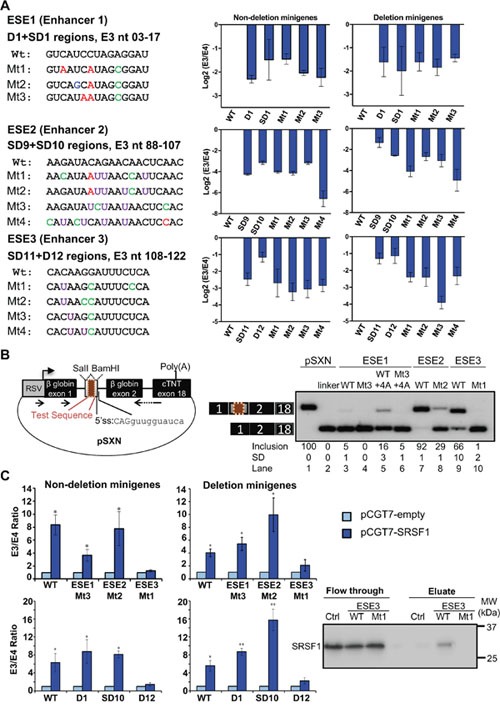
Confirmation of ESE1-3 as *bona fide BIM* E3 enhancers, and SRSF1 induction of E3 inclusion via ESE3 **(A)** Left, list of wild-type ESEs and the corresponding mutant sequences with their position relative to the first nucleotide of E3. Point mutations are highlighted in different colors. Right, real-time RT-PCR data for log 2 E3/E4 ratios for each construct, indicating that the mutations reduced the ratios to similar extend as the overlapping deletions. All deletions or point mutations are significant with P<0.05 compared to WT minigene using Student's t-test. **(B)** Left, diagram of the pSXN plasmid, in which test sequences (maroon box) were cloned into the indicated restriction sites in the gray exon. Arrows depict RT-PCR primers for detecting minigene transcripts. Right, RT-PCR data of K562 cells transfected with the indicated pSXN constructs. pSXN-13 with ESE (lane 1) and the pSXN-linker (lane 2) were respectively used as positive and negative controls. Exon inclusion reflects the enhancing effect of the inserted sequences (lanes 1, 3, 5, 7, 9), while absence or reduction of exon inclusion implies the disruption of the enhancer activity (lanes 2, 4, 6, 8, 10). Inclusion averages and Standard Deviations at the bottom were derived from at least three independent replicates (samples from different transfections). **(C)** Left, real-time RT-PCR data for E3/E4 ratios for ESE deletion and point mutant constructs co-transfected with pCGT7-empty or pCGT7-SRSF1 plasmids. All constructs except ESE3 D12 deletion and Mt1 point mutation show significant increase in E3/E4 ratio with SRSF1 overexpression (P<0.05 using Student's t-test). Note that the Y axis in this panel is not on Log 2 scale. Right, RNA pulldown and western blot shows binding of SRSF1 to ESE3 WT which is disrupted in Mt1. This is a representative western blot of three independent pulldowns.

We confirmed the regulatory activity of three identified ESEs, which we termed ESE1, ESE2 and ESE3 as ordered from 5’ to 3’ of E3 (Figure 1D and Figure 2A). To this end, we introduced point mutations which were not predicted to create any new *cis*-acting element by using Human Splicing Finder [63]. All these point mutations reduced *BIM* E3 splicing, mostly recapitulating the effects of the corresponding deletions, and further strengthening the evidence of these sequences as bona-fide ESEs. We next introduced these enhancer sequences in a heterologous exonic context, which is the weak alternative exon within pSXN minigene whose inclusion relies on an ESE (Figure 2B) [64]. While the control pSXN construct with the original ESE showed complete exon inclusion, removing this ESE only to leave a short linker results in complete skipping of this exon, as visualized by radioactive RT-PCR followed by PAGE (Figure 2B, lanes 1-2). Introduction of ESE1, ESE2 or ESE3 increased the inclusion of the pSXN exon compared to linker control, with ESE2 acting as the strongest enhancer (Figure 2B, lanes 3, 7 and 9). The constructs with the ESEs containing one of the point mutations tested above reduced the inclusion of this exon, consistent with these mutations disrupting the enhancers (Figure 2B, lanes 7-10). ESE1 was very weak in this context, which could be compensated by a point mutation adding another consensus nucleotide to the 5’ss (Wt+4A), and then further disrupted by mt3 (Figure 2, lanes 3-6). All in all, these point-mutation and heterologous context experiments strongly supported the three ESEs within *BIM* E3, with ESE2 as the strongest and ESE1 as weakest, and set the stage for testing of therapeutic ASOs to overcome *BIM* deletion resistance.

By co-transfecting an SRSF1-expression plasmid [65] together with the BIM Δ10 and Δ11 minigenes, we found that overexpression of this splicing factor largely increased E3 splicing (Figure 2C, Supplementary Figure 1C), indicating that these minigenes contain the SRSF1 responsive element. Indeed, while the minigenes with ESE1-2 deletions and point mutations responded to SRSF1 levels to a similar degree as wild-type minigenes, the deletion or mutation of ESE3 abolished the SRSF1 effects, indicating that ESE3 contains the SRSF1 responsive element. The *in silico tool* Human Splicing Finder [63] predicted the binding of SRSF1 to ESE3 (motif: CACAAGG) but not to mut 1 ESE3. Consistently, RNA pulldown showed SRSF1 binding to wild-type but not mut 1 ESE3, arguing that the SRSF1 promotion of E3 inclusion might be via direct binding to ESE3. Thus, our study also identified one *trans*-acting factor, which happens to be a proto-oncogene [32, 44], that regulates one of the three confirmed ESEs.

### Many splice-switching ASOs identified by coarse walk around *BIM* E3 in K562

**Figure 3:**
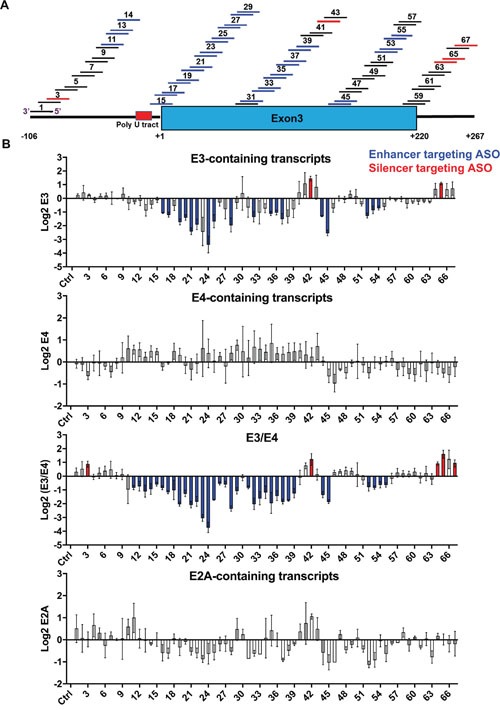
Coarse ASO walk to switch *BIM* splicing in K562 cells **(A)** Design of the 67 ASOs covering Intron 2 (left bold line), E3 (blue box) and intronic region downstream of E3 (right bold line). ASOs are shown as numbered short lines, in which the color depicts effects on *BIM* splicing: black denotes neutral ASOs which do not change splicing, blue indicates ASOs that increase E3/E4 ratio by likely targeting a splicing enhancer, and red indicates ASOs that decrease this ratio, likely blocking a silencer. The nucleotide coordinates relative to the 5’ end of E3 are indicated at the bottom. **(B)** Log 2 real-time RT-PCR data for transcripts containing E3, E4, and E2A, as well as the E3/E4 ratios. The ASOs 1-67 are ordered from left to right. Ctrl, control ASO with no target in human cells. ASOs that significantly alter the exon amounts relative to control are colored in blue or red as in panel A. All values derived from three independent transfection samples.

Next we performed a coarse ASO walk covering *BIM* E3 and flanking intronic regions (Figure 3A). We designed a total of 67 ASOs 18-nt long uniformly modified with 2’-O-methoxyethyl (MOE) nucleotides and a phosphorothioate backbone, and spaced by 5 nt from one another (5-nt walks, Supplementary Table 1). ASOs 1-14 covered the end of Intron 2, ASOs 15-60 covered E3 and ASOs 61-67 the region after E3. ASOs targeting the center of the polyU tract (with 17-21 Us) at the E3 3’ss were not synthesized because of the low specificity of this sequence.

We first tested all 67 ASOs in the K562 CML cell line, which lacks the *BIM* deletion allele and is thus sensitive to imatinib. Consistent with this notion, endogenous *BIM* transcripts show high E4 and lower E3/E4 ratios compared to the imatinib resistant cells, and E4 transcripts increase upon imatinib (Supplementary Figure 2A). Western blotting showed differences in BIMEL levels which correlated with imatinib reponses (Supplementary Figure 2B). Inclusion of E3, E4 and exon 2A (E2A) was measured by real-time RT-PCR upon ASO nucleofection. Most ASOs did not consistently alter the total levels of *BIM* mRNA as measured by E2A-containing transcripts (Figure 3B, bottom graph). As many as 20 ASOs significantly decreased E3 inclusion (Figure 3B, top graph, blue ASOs), and 34 ASOs also decreased the E3/E4 ratio (Figure 3B, third graph), which mapped to the end of Intron 2 (ASO 11-14), from the intron-exon junction to the middle of E3 (ASO 15-29, 31-39), and to the end of E3 including the poly(A) signal (ASO 44-45, 52-55). Even though these 34 ASOs decreased the E3/E4 ratio, none significantly increased E4 splicing (Figure 3B second graph), likely because E4 inclusion is already high in K562 (Supplementary Figure 2A). Finally, five ASOs significantly increased the E3/E4 ratio by targeting different regions of the transcript as shown in red (Figure 3B third graph, ASO 3, 42, 64, 65 and 67). These E3-activating ASOs likely target ISSs or ESSs, and have potential therapeutic applications for diseases with excessive BIM-dependent apoptosis. Nevertheless, this initial ASO walk identified numerous ASOs that reduce the E3/E4 ratio, with potential to resensitize resistant cells to TKIs.

### Imatinib and ASO treatment in KCL22 identifies the eight best ASOs that switch *BIM* splicing

The 39 ASOs that change *BIM* splicing (Supplementary Figure 3A) were next tested in the imatinib-resistant CML cell line KCL22, which is heterozygous for the 2,903-bp deletion allele, and expresses *BIM* transcripts with a higher E3/E4 ratio compared to K562 (Supplementary Figure 2A). From the 34 ASOs that decreased E3/E4 ratio in K562, only 2 did not do so in KCL22 cells (Supplementary Figure 3B, ASOs 11 and 12). Nevertheless, we did not detect a net increase in E4 levels by any of the 32 effective splice-switching ASOs. In addition, from the 5 ASOs that increased E3/E4 ratio in K562, only one did fail to do so in KCL22 (Supplementary Figure 3B, ASO 3). The splicing ratios upon ASO nucleofection in KCL22 were largely consistent with those in K562.

**Figure 4:**
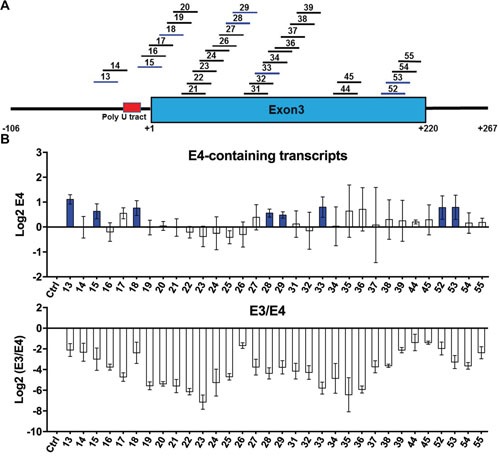
ASOs in KCL22 CML cell line after administration of imatinib **(A)** Summary of 32 shortlisted ASOs that decrease E3/E4 ratio (blue) in both K562 and KCL22. Format as in Figure 3A. **(B)** Log 2 real-time RT-PCR measurements of transcripts containing E4 as well as the E3/E4 ratios. Ctrl, control ASO. All tested ASOs reduce the E3/E4 ratio to different extents. ASOs that significantly increase the levels of E4 relative to control are colored in blue. All values derived from three independent transfection samples.

We next tested the 32 ASOs that decrease E3/E4 ratio (Figure 4A) in KCL22 cells treated with imatinib. This TKI directly inhibits the *BCR-ABL1* fusion oncogene by blocking its ATP-binding pocket and repressing its signaling [66, 67], thereby upregulating BIM at transcriptional and posttranslational levels to induce CML cell death [8, 12, 68]. However, in KCL22 cells, the imatinib-induced BIM upregulation is much smaller because of the deletion allele (Supplementary Figure 2), thus not enough to trigger apoptosis. Upon nucleofection of KCL22 cells, and in the presence of imatinib, all 32 ASOs strongly decreased *BIM* E3 and the E3/E4 ratio (Figure 4B and Supplementary Figure 4B). Remarkably, eight ASOs significantly increased total E4 levels which is necessary for these ASOs to restore functional BIM levels (as E4 encodes the pro-apoptotic BH3 domain). These ASOs mapped to the predicted BPS in Intron 2 for ASO 13, the Intron 2-E3 junction for ASO 15, the beginning of E3 for ASO 18, and two internal E3 regions for ASOs 28-29,33 and ASOs 52-53. These eight shortlisted ASOs were subsequently tested for *BIM* isoform profiling and effects on apoptosis.

### The eight shortlisted *BIM* ASOs enhance imatinib-mediated killing of KCL22 cells

**Figure 5:**
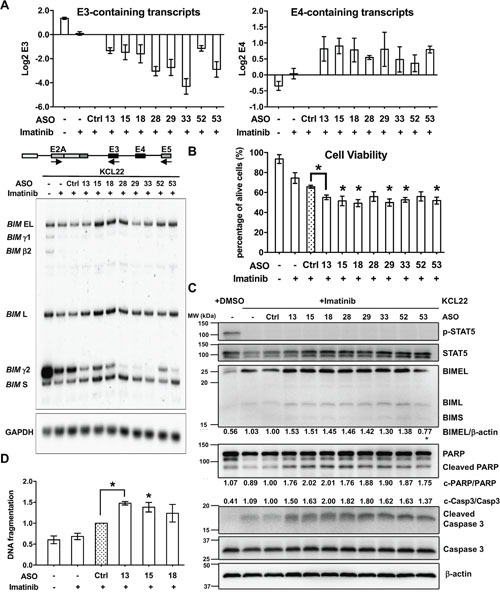
Detailed characterization of the effects of eight shortlisted ASOs in KCL22 cells treated with imatinib **(A)** Radioactive RT-PCR for *BIM* isoforms upon ASO nucleofection. As in the schematic, *BIM* RNA isoforms were amplified with a common forward primer in E2A and two reverse primers, one in exon 5 for E4-containing isoforms (EL, L and S) plus an isoform without E4 (β), and one in E3 for BIMγ (γ1 and γ2). Top graphs show Log 2 aggregate quantification of the bands for E3- and E4-containing isoforms. *GAPDH* RT-PCR was used as control for equal loading. **(B)** Cell viability assay by trypan blue in KCL22 cells treated with imatinib and ASOs. Graph shows the percentage of viable cells for each condition. Asterisks indicate significant difference between Ctrl and specific ASO treatments (P<0.05, Student's T Test). **(C)** Western blotting of imatinib- and ASO-treated KCL22 cells for markers of BCR-ABL1 signaling like phosphorylated (p-) STAT5 and total STAT5, as well as apoptotic markers like BH3-containing BIM isoforms, full-length and cleaved PARP and caspase 3. β-actin was used as loading control. This blot is representative of two experiments. Mean percentages indicate BIMEL levels normalized to β-actin, cleaved caspase 3 normalized to total caspase 3, and cleaved PARP normalized to total PARP. We did not see upregulation of BIMEL with ASO-53, but upregulation of cleaved caspase 3 and PARP were clear. **(D)** DNA fragmentation assay in KCL22 cells treated with 0.6 μM imatinib shows enhanced DNA fragmentation by ASO 13, 15 and 18.

Detailed tests of the eight shortlisted ASOs in imatinib-treated KCL22 cells by radioactive PCR [32] showed consistent results with the real time PCR (Figure 5A). The *BIM* E3-containing isoforms are divided into the predominant γ2 and the faint γ1, and both are downregulated upon imatinib treatment. All eight ASOs virtually abolished expression of γ1, and further reduced γ2, with ASOs 28, 29, 33 and 53 showing the strongest repression. In turn, all eight ASOs increased mRNA for BIML, and 15, 18, 28 and 53 also upregulated BIMEL and BIMS. By comparing the two merged E3-containing isoforms (*BIMγ* isoforms, with γ1 and γ2) and the three E4-containing isoforms (BIMEL, BIML and BIMS), we detected a clear and specific ASO-mediated downregulation of *BIMγ* and upregulation of BH3-containing *BIM* isoforms.

Next we assessed the effects of the eight shortlisted ASOs in cell viability, by measuring the percentage of dead cells by trypan blue (Figure 5B). Once again, real-time RT-PCR confirmed upregulation of E4-containing transcripts upon ASO treatment (Supplementary Figure 5A). Trypan blue exclusion revealed that imatinib decreased the number of live KCL22 cells very slightly, and control ASO further reduced it, suggesting unspecific yet mild toxic effects of the ASO nucleofection. Encouragingly, imatinib-treated KCL22 cells transfected with the eight ASOs showed higher levels of cell mortality, suggesting ASO-specific cell killing associated to *BIM* splicing switch. The ASOs enhanced imatinib-mediated cell killing by 9-16%, which is small but statistically significant. Bromodeoxyuridine pulse labeling experiments ruled out that imatinib and ASO-treated cells were arrested in any stage of the cell cycle (Supplementary Figure 6 and Supplementary Table 2). Furthermore, Western Blotting also confirmed the enhanced cell death triggered by the eight ASOs (Figure 5C). As expected, BCR-ABL1 signaling was effectively inhibited by imatinib treatment [69–72], as evidenced by decreased STAT5 phosphorylation. Nevertheless, the low levels of proapoptotic BIM associated to the deletion polymorphism render these cells largely resistant to cell death [19], and here we show that this resistance is reversed by the eight ASOs. We also detected higher cleaved caspase 3 and *Poly (ADP-ribose) polymerase* (*PARP*) as well as higher BIMEL and BIML isoforms associated with nucleofection of the shortlisted ASOs, consistent with enhanced apoptosis. As expected, imatinib-driven apoptosis in CML cells is induced by increased cleaved caspase 3 without a major change in total caspase 3 [73]. Furthermore, DNA fragmentation increased by three selected ASOs which were 13, 15 and 18 (Figure 5D). Altogether, these results show that the changes in *BIM* splicing by the eight effective ASOs also specifically induce imatinib-driven cell death in resistant CML cells.

### The eight shortlisted *BIM* ASOs enhance imatinib-mediated killing in *BIM* deletion-polymorphism containing K562 cells

**Figure 6:**
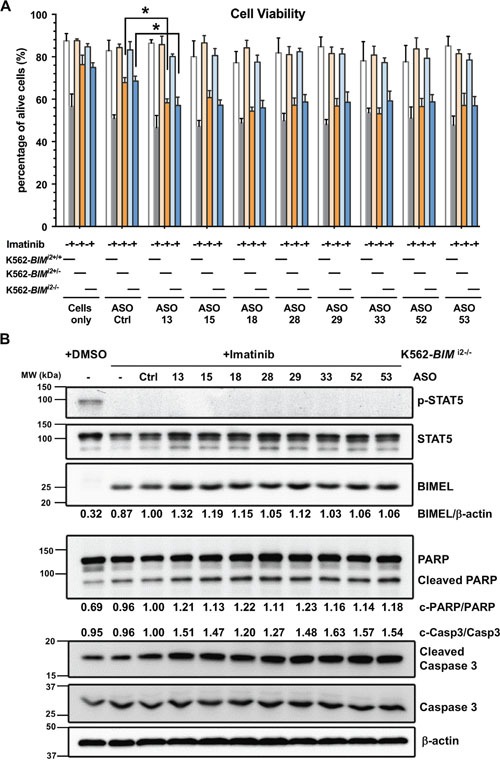
Analysis of shortlisted ASOs in edited K562 cells bearing the *BIM* deletion alleles and hence resistant to imatinib **(A)** Cell viability results for wild-type K562 (K562-*BIM*^I2+/+^), K562-*BIM*^I2+/-^ and K562-*BIM*^I2-/-^ cells treated with combinations of imatinib and ASOs. ASOs resensitize deletion bearing K562 cells to imatinib up to levels that are close to those for sensitive wild-type K562 cells. Asterisks indicate significant difference between Ctrl and specific ASO treatments (P<0.05, Student's T Test). **(B)** Western blotting for BCR-ABL1 signaling and apoptotic markers for K562-*BIM*^I2-/-^ cells plus imatinib and ASOs. This blot is representative of two experiments. We also indicated mean percentages of BIMEL and cleaved caspase 3 and PARP.

To validate our findings in KCL22 cells, we next tested the select ASOs in K562 clones which were rendered TKI-resistant by the presence of the *BIM* deletion polymorphism in either one (K562-*BIM*^I2+/-^) or two alleles (K562-*BIM*^I2-/-^) [19] (Figure 6). The number of deletion alleles corresponded with higher E3/E4 ratio compared to parental K562-*BIM*^I2+/+^ cells (Supplementary Figure 2). Compared to control ASO, the eight ASOs resensitized both K562-*BIM*^I2+/-^ and K562-*BIM*^I2-/-^ cells to imatinib-induced cell death (Figure 6A). Importantly, the cell death counts for these samples almost reached the values of imatinib-sensitive K562-*BIM*^I2+/+^ cells without the *BIM* deletion (compare gray bar in ‘Cell only’ or ASO-Ctrl to orange or blue bars for ASO-13-53). Thus, the imatinib-driven and ASO-enhanced cell mortality in resistant cells is not large but mimics what is seen in sensitive cells with imatinib alone, arguing for the biological effect of these ASOs. Some of these ASOs upregulated *BIM* E4-containing transcripts, except ASO-33 and perhaps others in K562-*BIM*^I2+/-^ (Supplementary Figure 5B). In addition, Western blotting of K562-*BIM*^I2-/-^ cells showed very clear increases in BIMEL (which contain the BH3 domain), as well as cleaved caspase 3 and cleaved PARP upon combined imatinib and *BIM* ASO treatment (Figure 6B). Overall, the results in the artificially induced imatinib-resistant K562-*BIM*^I2+/-^ and K562-*BIM*^I2-/-^ cells are consistent with the intrinsically-resistant KCL22 cell line. In conclusion, our data in K562 and KCL22 cells show that the eight ASOs switch *BIM* splicing, increase total levels of E4-containing isoforms, and also resensitize *BIM* deletion-containing CML cells to imatinib-induced apoptotic cell death.

## DISCUSSION

**Figure 7:**
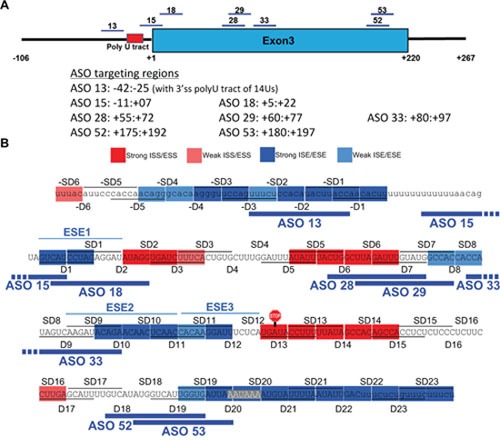
Summary of the map of *cis*-acting elements and effective splice-switching ASOs for *BIM* E3 splicing **(A)** Schematic mapping of the shortlisted ASOs which upregulate E4-containing transcripts and increase the imatinib-driven CML cell apoptosis. Their coordinates relative to the first nucleotide of E3 (+1) are also indicated. **(B)** Overlay of the map of splicing enhancers and silencers by deletion scan with the eight shortlisted ASOs, which mostly target ‘blue’ enhancers or other sequences with similar activity, such as BPS, 3’ss and polyadenylation signal.

This study shows that it is feasible to switch *BIM* splicing so as to enhance production of proapoptotic BIM isoforms, and thereby resensitize resistant *BIM* deletion-containing CML cell lines to imatinib. The eight most efficient splice-switching ASOs (Figure 7A) had similar effects in up to four different cell lines, including the naturally occuring imatinib-sensitive K562 (K562-*BIM*^I2+/+^) and the imatinib-resistant KCL22 cells, as well as two artificially-created K562 cell lines with the 2,903-bp deletion polymorphism [19]. The increase in E4-containing transcripts was accompanied with enhanced cell mortality and in apoptotic cell markers. We cannot exclude the possibility that the ASO-mediated cell mortality was diluted by efficient cell death which eliminated cells early on, but we did rule out a cell cycle arrest by *BIM* ASOs. Despite that some of the effects being small, the direct comparison of the imatinib plus ASO treatment between sensitive K562-*BIM*^I2+/+^ and either K562-*BIM*^I2+/-^ or K562-*BIM*^I2-/-^ cells suggests that these ASOs can reverse almost all resistance conferred by the Intron 2 deletion polymorphism. Nevertheless, we predict that further optimizations of ASO efficiency can be achieved by ‘microwalks’, which encompass small ASO sequence variations (produced by sliding the targeted sequences by one or few nucleotides) or slight changes in their length [74]. Perhaps ASO chemistries other than the 2’MOE or combinations of ASOs could also help enhance the ASO effects on apoptosis. In any case, these ASOs would only be used in the clinic in combination with imatinib or other TKIs, and perhaps with other emerging drugs such as BH3 mimetics or histone deacetylase inhibitors [75–77].

These eight ASOs should be further tested in primary tumors and especially in mouse models with BCR-ABL1 driven cancers [78]. The MOE-phosphorothioate chemistry confers ideal properties such as high specificity, molecular potency and resistance to degradation, while maintaining tolerability. Importantly, these ASOs are taken up by animal cells in the absence of a specific delivery agent by an as yet uncharacterized receptor-mediated endocytosis. In fact, ASOs can be systemically delivered in mice via either subcutaneous or intravenous injections, or via intrathecal injections for delivery into central nervous system, efficiently switching splicing in difficult cells like neurons [74, 79, 80]. Remarkably, ASOs were recently microinjected in utero for fetal delivery to mouse amniotic cavity, showing effects in the newborns even weeks after birth [81]. These recent developments in ASO delivery routes suggest that this technology might extend to cancer cells as well.

In the context of human cancers where the *BIM* deletion allele predicts poorer therapeutic responses and clinical outcomes, such as in CML or EGFR-NSCLC, the splice-switching ASOs we have identified may be used in combination with appropriate TKIs to overcome the negative effects of the *BIM* deletion. In addition, and in contrast to somatically acquired mechanisms of resistance which arise during the course of therapy, the germline nature of the *BIM* deletion would allow patients who harbor the deletion to be identified and treated when they first present. Furthermore, in the case of EGFR-NSCLC where tumor tissue may be hard to obtain, genotyping for the *BIM* deletion may be performed on normal tissue, e.g from peripheral blood mononuclear cells or even a buccal swab. Because other drugs have also been described to overcome the effects of the *BIM* deletion, including so-called BH3 mimetic drugs and histone deacetylase inhibitors, it may also be possible to combine splice-switching ASOs with either of these agents to further enhance their effects, although this might be at the risk of increased toxicity [19, 55–58].

In addition to providing proof-of-concept for splice-switching ASOs, this study also presents new mechanistic insights into the alternative splicing regulation of an important proapoptotic factor. Our deletion scan in the *BIM* minigenes revealed a number of regulatory E3 *cis*-acting splicing elements. Notably, some of the effective ASOs that reduced the E3/E4 ratio indeed map to ESEs and other intronic or exonic elements important for E3 inclusion, such as the predicted BPS and the Intron 2-E3 junction as part of the 3’ss (Figure 7B). The BPS and 3’ss targeting ASO-13 and ASO-15 very likely abolish recognition by the canonical splicing factors such as U2 snRNP and U2-auxiliary factor (U2AF) heterodimer, respectively. Furthermore, the disruptive effects of deletions at the polyadenylation signal and neighboring sequences on E3 inclusion reflect the synergistic stimulation between 3’-end formation and splicing [60–62]. The canonical AAUAAA polyadenylation signal was only very recently proven to be bound by the Cleavage and Polyadenylation Specificity Factor (CPSF) subunits CPSF30 and Wdr33, instead of CPSF160 [82]. Mutations in the polyadenylation signal repress *in vitro* splicing of the last intron [62]. The synergistic stimulation of splicing and 3’-end formation could be attributed to the interactions between U2 snRNP and CPSF [60], or between U2AF65 and Cleavage Factor Im 59KDa [61]. Last, splicing and 3’-end formation are co-transcriptionally coupled as also characterized in cell-free extracts [83, 84]. The ASOs targeting the polyadenylation signal and downstream sequences did not efficiently switch *BIM* splicing, probably because of the AU richness of these target sequences may lead to inefficient recognition, or to diversion of ASO binding to other AU-rich sequences. Notwithstanding this, ASO-52 and ASO-53, targeting more GC-rich sequences upstream of the AAUAAA in E3, switched splicing and increased imatinib-driven apoptosis, likely by interfering with CPSF binding.

ESE1-3 were further supported by point mutations also reducing the E3/E4 ratio, and by their splicing enhancer activity recapitulated in the heterologous pSXN context [64]. Direct comparison of these three ESEs in pSXN revealed their intrinsic strengths, showing that ESE2 is the strongest while ESE1 is very weak in this context, despite that ESE1-targeting ASO-18 showed optimal effects in splicing and imatinib-induced cell death. The apparent weakness of ESE1 in pSXN could be due to its context dependency, as its very close proximity to the 3’ss may facilitate efficient enhancement of *BIM* E3 inclusion. Alternatively, insertion of ESE1 into the pSXN internal exon may have created a new junction with silencing activity, or its proximity to the 5’ss of this small exon sterically reduced U1 binding, as U1 snRNP footprint extends within the upstream exon far beyond the three conserved nucleotides [85]. Importantly, these ESEs show the same activity in both deletion and non-deletion polymorphism minigenes, indicating that they act independently of the multiple and redundant silencers within the 2,903-nt fragment [31]. From the eight shortlisted ASOs, only ASO-33 partially overlaps with ESE2, while ASO-34-39 targeting ESE2 and ESE3 did show splicing switch, yet without clear upregulation of E4-containing transcripts. In fact, the mechanisms by which a majority of ASOs switch splicing by repressing E3 inclusion but not increasing E4 levels, remain to be elucidated. Last, ASO-28 appears to target a silencer region yet shows clear reduction of E3/E4 levels and increased imatinib-driven cell death, suggesting that the enhancers and silencers cannot be mapped with precision by the deletion scan, and/or that these silencers might act by complex recruitment of both activators and repressors.

An added value of this study is the identification of ESE3 as the sequence that mediates the increase in E3 inclusion by the SRSF1 splicing factor and protooncogene, which was not known before [32]. This result further confirms our identified enhancers and suggests that ESE3 might mediate TKI resistance in tumors with high levels of SRSF1. Despite that our RNA pulldowns are consistent with direct binding of SRSF1 to ESE3, only future *in vitro* splicing experiments in cell-free extracts might confirm such direct effects, which should reveal further mechanistic insight into this medically relevant alternative splicing event.

The richness of *cis*-acting elements regulating E3 splicing has been seen in other constitutive and alternative exons [59, 86]. Indeed, a recent mass-mutational study within an exon revealed that point mutations at 90% of exonic positions changed the splicing patterns while many combinations of double mutations exhibited non-additive effects [87]. This study revealed the complex relation between an exonic sequence and its splicing pattern, and strikingly, demonstrated that virtually each exonic nucleotide bears splicing regulatory information. Nevertheless, our combination of systematic deletions and ASO walking mostly confirms the splice-switching action of the ASOs, illustrates that deletion scans can identify functional regions for initial ASO targeting, but that other approaches are required to identify optimal ASOs.

In summary, our study not only expands our knowledge of the regulatory mechanisms for a medically important alternative splicing event, but also reveals a few ASOs as potential adjuvant drugs to overcome TKI resistance in CML and other tyrosine kinase-driven cancers, as well as personalized therapeutics for patients with the *BIM* deletion polymorphism.

## MATERIALS AND METHODS

### Cell lines and chemicals

We cultured the K562 and KCL22 cell lines in Roswell Park Memorial Institute (RPMI) 1640 Medium (Hyclone) supplemented with penicillin/streptomycin (Gibco) and 10% fetal bovine serum (Gibco) at 37°C with 5% CO_2_. We maintained the edited K562-*BIM*^I2+/-^ and K562-*BIM*^I2-/-^ cells [19] in RMPI-1640 medium supplemented with penicillin/streptomycin and 20% fetal bovine serum. We dissolved imatinib in DMSO at 50%, stored it at -20°C, and used it at 2 μM for all experiments unless otherwise indicated. We obtained 2’O-methoxyethyl (MOE) and phosphorothioate ASOs from IONIS Pharmaceuticals (Carlsbad, CA). We dissolved ASOs in nuclease-free H_2_O and kept them at -20°C.

### Plasmid construction

We made mutant plasmids in the context of both *BIM* Δ10 and Δ11 minigenes [31], using PCR mutagenesis with specific primers (sequences available upon request) and KAPA HiFi DNA polymerase (KAPA Biosystems). We generated serial deletions and staggered deletions of 10 nucleotides each in *BIM* E3 and upstream 106 nt intronic region, by using specific primers which contained the flanking regions of the deleted sequence. We introduced point mutations in enhancer sequences of *BIM* E3 using specific primers. pCGT7-empty and pCGT7-SRSF1 plasmids were gifted by Prof Javier F Cáceres from MRC Human Genetics Unit at Edinburgh, UK [65].

The pSXN plasmidwas provided by Prof Thomas A Cooper from Baylor College of Medicine, USA [64]. We annealed equimolar amounts of a pair of complementary DNA oligonucleotides containing designed test sequences and restriction overhangs by heating at 95°C for 5 min followed by gradual cooling to room temperature for 1 h. We then 5′-end phosphorylated the annealed oligonucleotides by T4 polynucleotide kinase (T4PNK; New England Biolabs) in 1x ligation buffer, and ligated the duplex into the *Bam*HI and *Sal*I sites in the alternative exon within pSXN13 vector.

### Semi-quantitative PCR

We labeled the 5′-end of 10 pmol forward primer using 10U T4 PNK (New England Biolabs) and 10.2 pmol γ-32P-ATP (60 μCi; Perkin Elmer). We then purified the labeled primers by illustra Microspin G-25 column (GE Healthcare Life Sciences) and added them to the primer mixture containing 100 pmol unlabeled reverse primer and 90 pmol unlabeled forward primer. We used cDNA for PCR-amplification using Go-Taq polymerase (Promega) with the radiolabeled primer mixture for 22 cycles. We separated the PCR products by 8% (w/v) native polyacrylamide gel, and subsequently exposed the gel's radioactive signals to storage phosphor-imaging screen which was read by Typhoon Trio variable mode imager (GE Healthcare Life Sciences). We analyzed the band intensity with ImageQuant TL software (GE Healthcare Life Sciences). We generated gel images by exposing gels to X-ray films (Kodak) for 12-48 h in X-ray cassette at -80°C and then processed images by Kodak Model 2000 X-12 ray film processor. The average exon inclusion and standard deviation were derived from the band intensities from at least three experimental replicas. The splicing pattern from endogenous *BIM* transcripts were normalized to *GAPDH*. The following primers were used: pSXN_βglobinEx1-F: 5’-AGGTGAACGTG GATGAAGTTGGTGGTG-3’; pSXN_βglobinEx2-R: 5’-CGTGCAGCCTTTGACCTAC TAGTGTG-3’; BIM-E2A-F: 5′-ATGGCAAAGCAACCTTCTGATG-3′, BIM-E3-R: 5′-ATGGTGGTGGCCATACAAAT-3′; BIM-E5-R: 5’-TAACCATTCGTGGGTGGTCT-3’.

### Quantitative real-time PCR

We isolated total RNA from cells using PureLink RNA mini kit and treated it with TURBO DNase (both from Life Technologies). We reverse transcribed 1 μg total RNA with M-MuLV reverse transcriptase (New England Biolabs) and oligo-dT. We performed real-time PCR in a 20 μl mixture containing 2 μl of the five-fold diluted cDNA, 10 μl SYBR Select Master Mix (Life Technologies) and 200 nM of each primer in the CFX96 Real-Time PCR System (Bio-Rad) using the following parameters: 95°C for 3 min, 40 cycles of 95°C for 10 sec, 58°C for 30 sec and 72°C for 20 sec. We calculated the fluorescence threshold value (Ct) using the thermocycler system software. We ruled out nonspecific products by both the analysis of the melting curves and by electrophoresis in 2% agarose gels. We normalized endogenous and minigene transcripts to β-actin or adenovirus exonic sequence (U) transcript levels, respectively. We used the following primers: β-actin (forward: 5’-CCA GAGGCGTACAGGGATAG-3’; reverse: 5’-CCAACCGCGAGAAGATGA-3’), SRSF1 (forward: 5’-TTCTACAAATACGGCGCTATCC; reverse: 5’-GTACCCATCGTAATCATAGCCG) *BIM* E2A (forward: 5’-TTCCCCCAAATGTCTGACTC-3’; reverse: 5′-CTTGTGGCTCTGT CTGTAGGG-3′), *BIM* E3 (forward: 5’-CCAGGCCTTCAACCACTATC-3′; reverse: 5′-ATGGTGGTGGCCATACAAAT-3′), *BIM* E4 (forward: 5’-TTCCATGAGGCAGGCTG AAC-3′; reverse: 5′-CCTCCTTGCATAGTAAGCGTT-3′), U (forward: 5′-CGAGCTCA CTCTCTTCCGC-3′; reverse: 5′-CTGGTAGGGTACCTCGCA-3′), U-E3 (forward: 5′-C GAGCTCACTCTCTTCCGC-3′; reverse: 5′-CTCTAGGATGACTACTGGTAGGGT-3′ or U-E3-2 (forward: 5′-CTGCGAGGTACCCTACCAGT-3′; reverse: 5’- GGTGGTGG CCATACAAATCT-3’ which were used for detection of E3 levels in D1, SD1, D2 and ESE1 mutants), U-E4 (forward: 5′-CGAGCTCACTCTCTTCCGC-3′; reverse: 5′-CCTCATGGAAGCT GGTAGGGT-3′). Thus, the U-E3, U-E3-2 and U-E4 are junction primers that enhance specificity of amplification. We expressed values as fold change over the corresponding values for the control by the 2^-ΔΔCt^ method. Graphs represent the Log 2 of the corresponding real-time RT-PCR values or ratios unless otherwise stated.

### Transfection

We transiently nucleofected 1×10^6^ K562 or KCL22 cells with 2 μM ASO using SF Cell Line Nucleofector kit (Lonza) with program FF-120 and EH-198, respectively. We harvested cells 48 h after nucleofection. For the samples with Imatinib treatment, we first nucleofected cells and after 18 h added Imatinib, and collected cells 48 h later.

We transfected K562 cells with minigenes using Xtreme GENE HP transfection reagent (Roche Applied Science) according to the manufacturer's protocol. We incubated the transfected cells at 37°C with 5% CO_2_ for 48 h before RNA/protein extraction. For overexpression assay, we mixed minigene plasmids with control pCGT7-empty or pCGT7-SRSF1 plasmids at a 1:1 ratio.

### Definition of enhancers and silencers from deletion scan

For each deletion (D) or staggered deletion (SD) in 10 nt segments, the E3/E4 ratio is considered as the average of the mean E3/E4 ratios from both Δ10 and Δ11 minigenes. To derive the final map (Figure 1D), we took the average E3/E4 ratio between each D and SD deletion in both Δ10 and Δ11 (total of 4 values) and applied it to the 5 nt region where they overlapped. We highlighted each segment as (i) enhancer in blue, (ii) silencer in red, or (iii) neutral as uncolored, based on the following criteria: (i) the 5 nt sequence is part of an ESE/ISE if its E3/E4 average shows a ≥30 % decrease compared to WT; (ii) the 5 nt sequence is part of an ESS/ISS if the corresponding average shows a ≥30% compared to WT. For strong ESE/ISE or ESS/ISS, the cutoff is a ≥50% decrease or increase in the mean E3/E4 ratio compared to WT, respectively.

### RNA pulldown

We performed RNA pulldown using Pierce Magnetic RNA-Protein Pull-down kit (USA). We incubated 50 pmol synthetic RNA (IDT, USA) with 120 μg HeLa nuclear extract. We used the following RNA oligos: (ESE3 WT 5’-UCAACCACAAGGAUUUCUCAUGAUA-3’ and ESE3 Mt1 5’-UCAACCAUAAGCAUUUCCCAUGAUA-3’). We separated the pulldown eluates and flow through by 12% SDS-PAGE, followed by Western Blotting. We performed the pulldown assay in triplicate with consistent results.

### Western blotting

After PBS washing, we resuspended cells in ice-cold Radioimmunoprecipitation assay (*RIPA*) lysis buffer (Millipore) supplemented with proteinase inhibitors and phosphatase inhibitors (Roche). We centrifuged cell lysates at 14,000 rpm for 30 min at 4°C, and estimated the protein concentrations with the Bradford assay (Bio-Rad). We resolved 10-20 μg of protein samples by 12.5-15% SDS-PAGE gels and then transferred them to PVDF membrane (Bio-Rad). We incubated the blocked membranes with primary and HRP-tagged secondary antibody in 5% (w/v) nonfat milk and Tris buffered saline-Tween (TBST). Primary antibodies were: anti-SRSF1 (gifted by Prof Adrian R Krainer from Cold Spring Harbor Laboratory), mouse monoclonal anti-human β-actin (#AC-15, Sigma); rabbit polyclonal anti human BIM (#2819), Caspase-3 (#9662), Cleaved Caspase-3 (#9661), PARP (#9542), phosph-STAT5 (#9359) and STAT5 (#9363) antibodies, from Cell Signaling Technology. Secondary antibodies include anti-mouse IgG or anti-rabbit IgG HRP linked antibodies (Santa Cruz). We detected immunostained bands with the enhanced chemiluminescence substrate (Perkin-Elmer) exposed to LAS-4000 imager (Fujifilm).

### Viable cell counting

We counted viable cells through Trypan Blue staining (Sigma-Aldrich) with a haemocytometer (Hausser Scientific), 48 h after TKI treatment.

### ELISA-based DNA fragmentation assay

Upon harvesting cells at indicated time points, we detected mono- and oligo-nucleosomes in the apoptotic cells using the Cell Death Detection ELISA (Sigma-Aldrich), according to the manufacturer's instructions.

### Cell cycle analysis

We analyzed the cell cycle kinetics using Apoptosis, DNA damage and Cell Proliferation kit (BD Biosciences). In brief, we treated KCL22 cells with 10 μM Bromodeoxyuridine (BrdU), and after 4 h, we fixed the cells and treated them with DNase according to the manufacturer's instructions. After staining with PerCP-Cy^TM^5.5 Mouse Anti-BrdU and DAPI provided in the kit, we analyzed the cells using LSRFortessa Cell Analyzer (BD Biosciences).

### Statistical analysis

We obtained all the data used for statistics analysis from three independent experiments (different samples from different transfections or treatments). We assessed the difference between groups by the two-tailed Student's t-test using the Microsoft Excel software, with indicated significance at *P*≤ 0.05.

## SUPPLEMENTARY MATERIALS FIGURES AND TABLES





## Abbreviations

5’ss: 5’ splice site
3’ss: 3’ splice site
ASO: antisense oligonucleotide
BCL2L11: B-Cell Lymphoma 2-like 11
BIM: BCL-2 interacting mediator of cell death
BIMEL: BIM extra large
BIML: BIM large
BIMS: BIM small
BIMγ: BIM gamma
bp: base pair
BPS: branch point sequence
BrdU: bromodeoxyuridine
CML: chronic myeloid leukemia
CPSF: Cleavage and Polyadenylation Specificity Factor
E2a: exon 2a
E3: exon 3
E4: exon 4
EGFR: Epidermal growth factor receptor
ESE: exonic splicing enhancer
ESS: exonic splicing silencer
D: deletion
hnRNP: heterogeneous nuclear ribonucleoprotein
ISE: intronic splicing enhancer
ISS: intronic splicing silencer
MDS: myelodysplastic syndromes
MOE: 2’-O-methoxyethyl
NSCLC: non-small cell lung cancer
nt: nucleotide
OS: overall survival
PAGE: polyacrylamide gel electrophoresis
*PARP*: *poly (ADP-ribose) polymerase*
PFS: progression free survival
PNK: polynucleotide kinase
*RIPA*: radioimmunoprecipitation assay
RPMI: Roswell Park Memorial Institute
RT-PCR: reverse-transcription polymerase chain reaction
SD: staggered deletion
SDS: sodium dodecyl sulfate
snRNP: small nuclear ribonucleoprotein
SR: serine/arginine-rich proteins
SRSF1: serine/arginine-rich splicing factor 1
STAT5: signal transducer and activator of transcription 5
TKI: tyrosine kinase inhibitor
U2AF: U2-auxiliary factor
WT: wild type
