# Supplementary material for: Overcoming imatinib resistance conferred by the BIM deletion polymorphism in chronic myeloid leukemia with splice-switching antisense oligonucleotides
Source: Oncotarget. 2017 Sep 6;8(44):77567–85. doi: 10.18632/oncotarget.20658 (PMC5652800; doi:10.18632/oncotarget.20658)
Supplement: Supplementary file 1 [file oncotarget-08-77567-s001.pdf]

Overcoming imatinib resistance conferred by the *BIM* deletion polymorphism in chronic myeloid leukemia with splice-switching antisense oligonucleotides

SUPPLEMENTARY MATERIALS

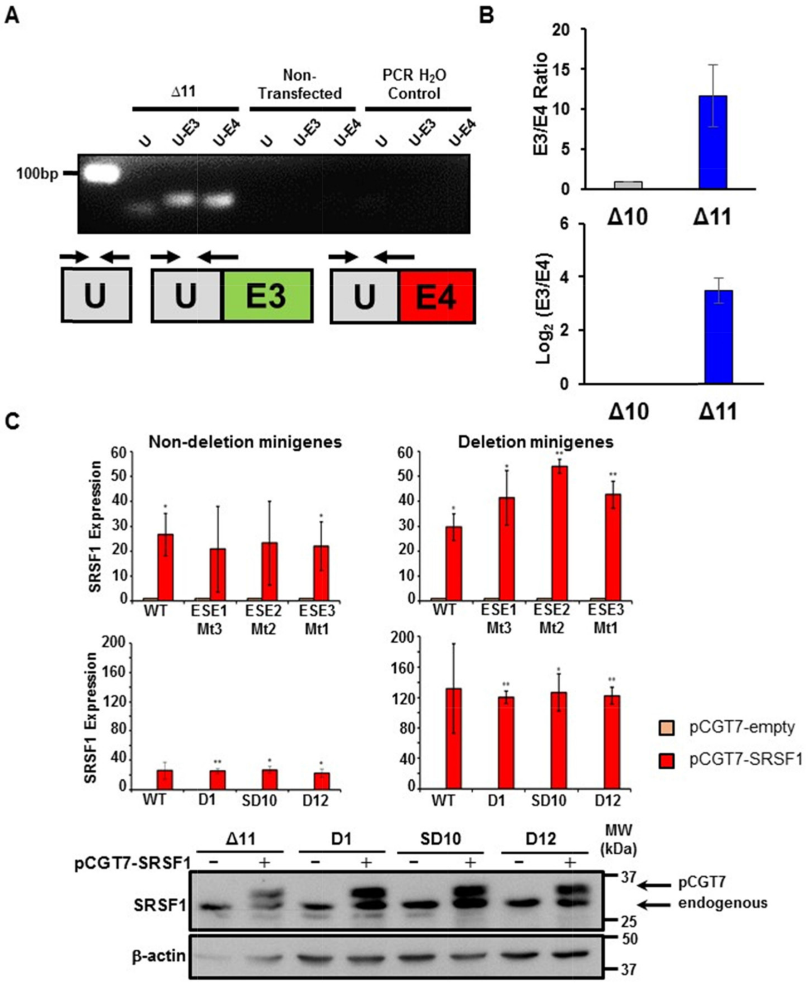

Supplementary Figure 1: Specificity of the primers for real-time RT-PCR analysis of *BIM* minigene transcripts, and SRSF1 experiments. **(A)** RT-PCR analysis of U-, E3-, and E4-containing transcripts from K562 cells transfected with Δ11 (deletion minigene) or non-transfected cells. Negative control PCR has water as template. Arrows depict the location of the primers: The forward U primer is paired with either a U, U-E3 and U-E4 reverse primers, with the two latter spanning the junction of E3 or E4 with U, respectively. RT-PCR for Δ11 but not for untransfected or water control produced single bands with the sequence of the expected splice pattern. Δ10 (nondeletion minigene) analysis showed the same result (data not shown). **(B)** Real-time RT-PCR data for E3/E4 ratios for Δ10 or Δ11. We plotted the ratios directly on top, and as Log2 at the bottom, which was used throughout the manuscript. **(C)** Real-time RT-PCR measurements of the relative SRSF1 transcript levels (normalized to β-actin) from K562 cells cotransfected with ESE deletion/point mutant constructs and pCGT7-empty or pCGT7-SRSF1 plasmids. Note that the mRNA expression levels of SRSF1 greatly varied among each series of experiments but had comparable means within each series, due to different transfection efficiencies and other issues. Despite all these differences, the ESE3 deletions and point mutations always responded to much lesser extent or not at all to SRSF1 over-expression, when compared to all the wild-type or other mutant minigenes. Bottom, representative western blot showing consistent overexpression of SRSF1 in all samples of K562 cells cotransfected with *BIM* minigenes and pcGT7-empty or pCGT7-SRSF1 plasmids.

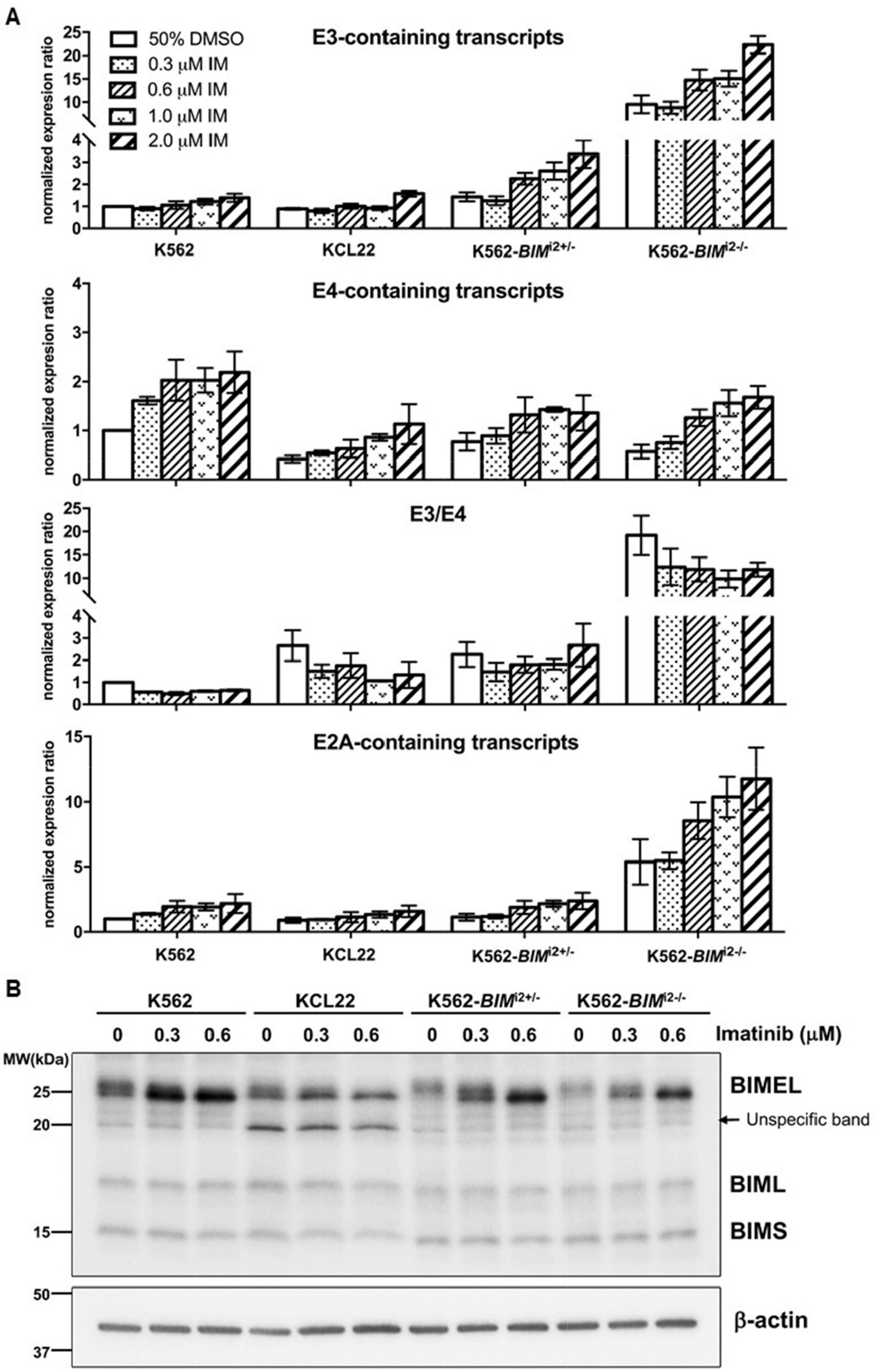

Supplementary Figure 2: Endogenous expression of *BIM* exon-specific transcripts and protein isoforms in CML cell lines. **(A)** RNA levels of each *BIM* exon-specific transcripts and exon ratios in four cell lines 24 h after treatment with different doses of imatinib, measured by real-time RT-PCR. We normalized the levels of each exon to β-actin. All values derived from three independent experiments. **(B)** Western blotting detection of BIM isoforms in cell lines treated with different amounts of imatinib, with β-actin as loading control. Gel is representative of three independent experiments.

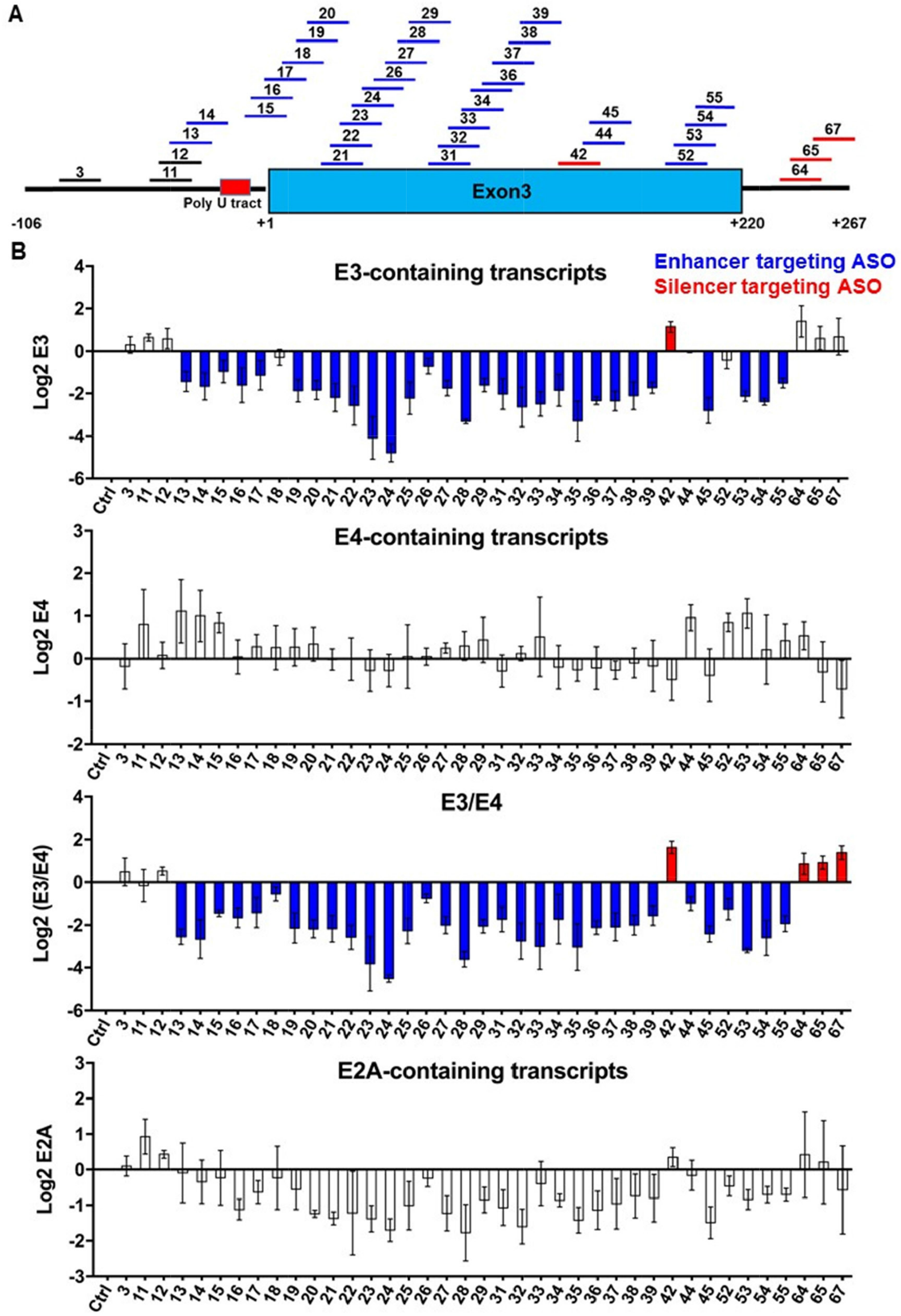

Supplementary Figure 3: Short-listed *BIM* ASOs in KCL22 CML cell line which is resistant to imatinib. (A) Summary of shortlisted ASOs, including 34 ASOs that decrease E3/E4 ratio (blue) and 5 ASOs that increase this ratio (red). Format as in Figure 3A. **(B)** Log 2 real-time RT-PCR measurements of transcripts containing E3, E4, and E2A, as well as E3/E4 ratios. Ctrl, control ASO. ASOs that significantly alter the exon amounts relative to control are colored in blue or red. All values derived from three independent transfection samples.

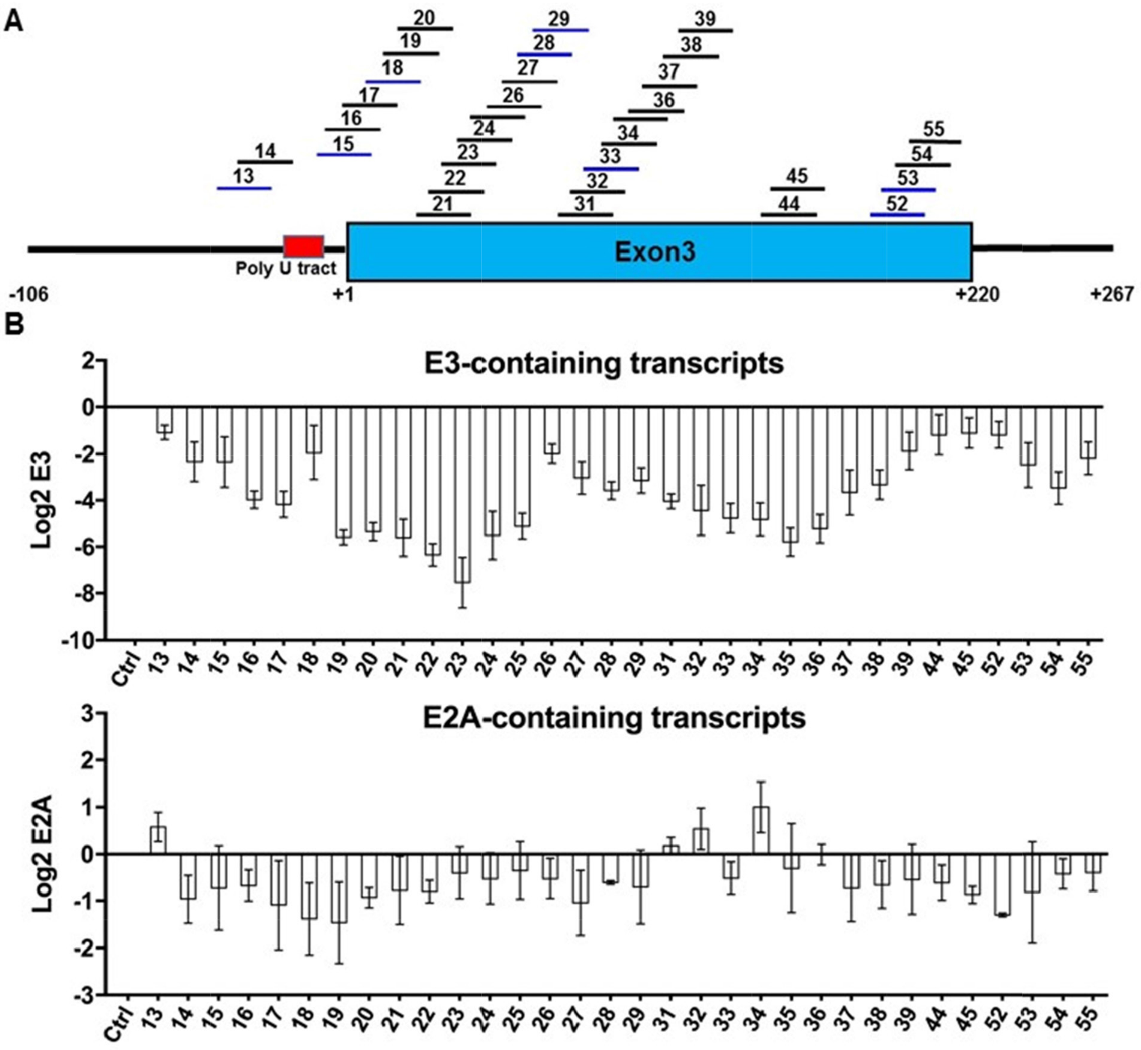

Supplementary Figure 4: ASOs in KCL22 CML cell line after administration of imatinib. (A) Summary of 32 shortlisted ASOs that decrease E3/E4 ratio (blue) in both K562 and KCL22. Format as in Figure 3A. **(B)** Log 2 real-time RT-PCR measurements of transcripts containing E3 and E2A. Ctrl, control ASO. All tested ASOs reduce E3 inclusion to different extents. All values derived from three independent transfection samples.

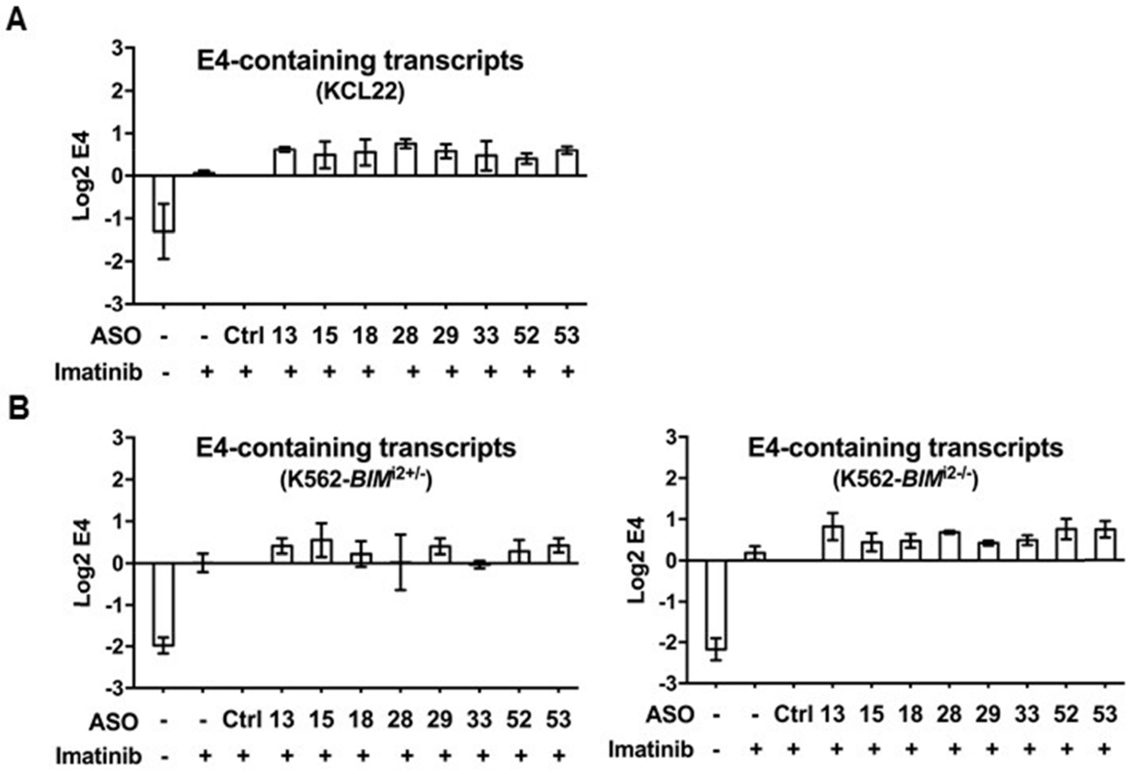

Supplementary Figure 5: Detailed characterization of the effects of eight shortlisted ASOs in KCL22 and K562 cells treated with imatinib. **(A)** The E4-containing transcripts (Log 2) measured by real time RT-PCR in KCL22 cells treated with imatinib and ASOs confirm effective splice-switching. **(B)** Imatinib and *BIM* ASO-driven upregulation of E4-containing transcripts (Log 2 real time RT-PCR) in K562-*BIM*$^{i2+/-}$ and K562-*BIM*$^{i2-/-}$ cells.

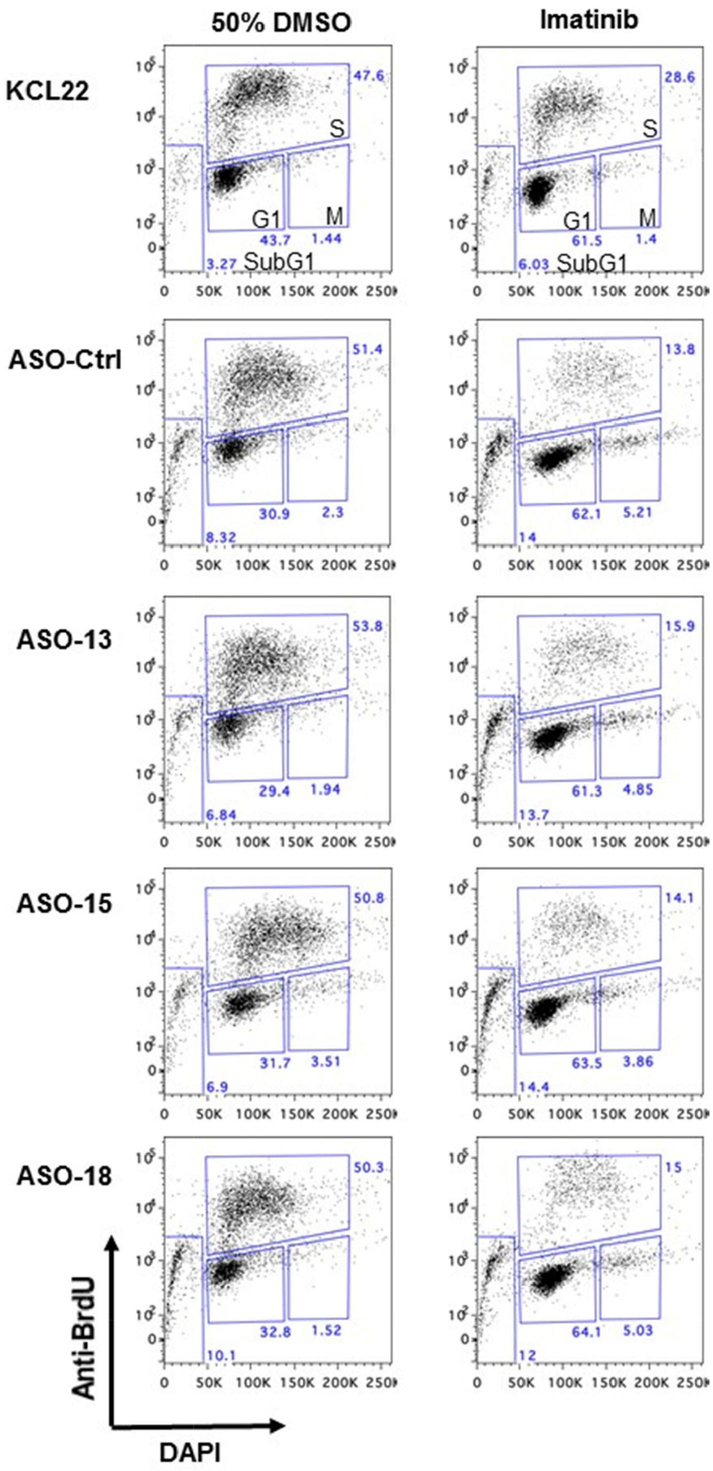

Supplementary Figure 6: Bromodeoxyuridine (BrdU) pulse chase experiment in KCL22 cells treated without (DMSO) or with 0.6 µM imatinib and ASOs. Plots display data that are representative of three independent experiments.

Supplementary Table 1: List of splice-switching *BIM* ASOs

See Supplementary File 1

Supplementary Table 2: Percentage of KCL22 cells with indicated treatments in different cell cycle stages after BrdU pulse chase assay

(%)	50% DMSO					Imatinib				
	KCL22	ASO-Ctrl	ASO-13	ASO-15	ASO-18	KCL22	ASO-Ctrl	ASO-13	ASO-15	ASO-18
G1	43.73±1.85	34.60±4.86	32.33±4.01	32.43±2.19	31.53±3.10	62.10±1.59	62.63±5.52	59.03±3.67	59.67±4.38	60.60±5.89
S	46.73±2.32	45.23±5.96	46.73±7.66	44.47±8.81	44.87±5.35	26.83±2.11	11.83±2.07	14.50±1.71	13.23±1.71	14.17±0.85
G2/M	2.07± 0.54	4.60± 2.19	4.50± 2.51	6.01± 3.89	5.72± 3.65	2.35± 0.95	7.47± 1.99	7.49± 2.45	6.93± 3.07	7.58±3.08
SubG1	2.75± 0.99	7.54± 1.81	7.28± 0.75	6.95± 1.32	7.28± 2.46	5.43± 0.90	12.14±3.94	11.90±1.61	13.03±1.95	10.66±1.16

We show values as mean±sd for three independent experiments.
