# Supplementary material for: Overcoming imatinib resistance conferred by the BIM deletion polymorphism in chronic myeloid leukemia with splice-switching antisense oligonucleotides
Source: Oncotarget. 2017 Sep 6;8(44):77567–85. doi: 10.18632/oncotarget.20658 (PMC5652800; doi:10.18632/oncotarget.20658)
Supplement: Supplementary file 2 [file oncotarget-08-77567-s002.docx]

**Supplementary Table 1: List of splice-switching *BIM* ASOs**

| **ASO number^** | **Sequence (5’ to 3’)** | **Location*** |
| --- | --- | --- |
| ASO-1 | AGCCTCTATGGAGAACAG | -102 : -85 |
| ASO-2 | GGCACAGCCTCTATGGAG | -97 : -80 |
| ASO-3 | AAAATGGCACAGCCTCTA | -92 : -75 |
| ASO-4 | AATGTAAAATGGCACAGC | -87 : -70 |
| ASO-5 | GTGGGAATGTAAAATGGC | -82 : -65 |
| ASO-6 | TGTTGGTGGGAATGTAAA | -77 : -60 |
| ASO-7 | TGCCCTGTTGGTGGGAAT | -72 : -55 |
| ASO-8 | CCTTGTGCCCTGTTGGTG | -67 : -50 |
| ASO-9 | GGAACCCTTGTGCCCTGT | -62 : -45 |
| ASO-10 | AAACTGGAACCCTTGTGC | -57 : -40 |
| ASO-11 | TGGAGAAACTGGAACCCT | -52 : -35 |
| ASO-12 | GTATGTGGAGAAACTGGA | -47 : -30 |
| **ASO-13** | **GGTAAGTATGTGGAGAAA** | **-42 : -25** |
| ASO-14 | GTGTTGGTAAGTATGTGG | -37 : -20 |
| **ASO-15** | **ATGACTACTGTTAAAAAA** | **-11 : +07** |
| ASO-16 | CTAGGATGACTACTGTTA | -06 : +12 |
| ASO-17 | ATCCTCTAGGATGACTAC | -01 : +17 |
| **ASO-18** | **CCTATATCCTCTAGGATG** | **+5 : +22** |
| ASO-19 | GATCACCTATATCCTCTA | +10 : +27 |
| ASO-20 | TGAAAGATCACCTATATC | +15 : +32 |
| ASO-21 | CACAGTGAAAGATCACCT | +20 : +37 |
| ASO-22 | CAAAGCACAGTGAAAGAT | +25 : +42 |
| ASO-23 | AAATCCAAAGCACAGTGA | +30 : +47 |
| ASO-24 | AATATAAATCCAAAGCAC | +35 : +52 |
| ASO-25 | CAGTAAATATAAATCCAA | +40 : +57 |
| ASO-26 | TAAGCCAGTAAATATAAA | +45 : +62 |
| ASO-27 | AAATCTAAGCCAGTAAAT | +50 : +67 |
| **ASO-28** | **CATACAAATCTAAGCCAG** | **+55 : +72** |
| **ASO-29** | **GTGGCCATACAAATCTAA** | **+60 : +77** |
| ASO-30 | TGGTGGTGGCCATACAAA | +65 : +82 |
| ASO-31 | GACTATGGTGGTGGCCAT | +70 : +87 |
| ASO-32 | ATCTTGACTATGGTGGTG | +75 : +92 |
| **ASO-33** | **TCTGTATCTTGACTATGG** | **+80 : +97** |
| ASO-34 | GTTGTTCTGTATCTTGAC | +85 : +102 |
| ASO-35 | GTTGAGTTGTTCTGTATC | +90 : +107 |
| ASO-36 | TTGTGGTTGAGTTGTTCT | +95 : +112 |
| ASO-37 | AATCCTTGTGGTTGAGTT | +100 : +117 |
| ASO-38 | TGAGAAATCCTTGTGGTT | +105 : +122 |
| ASO-39 | TATCATGAGAAATCCTTG | +110 : +127 |
| ASO-40 | AAAGGTATCATGAGAAAT | +115 : +132 |
| ASO-41 | TATAAAAAGGTATCATGA | +120 : +137 |
| ASO-42 | GTGGCTATAAAAAGGTAT | +125 : +142 |
| ASO-43 | TGGCTGTGGCTATAAAAA | +130 : +147 |
| ASO-44 | AGAGGTGGCTGTGGCTAT | +135 : +152 |
| ASO-45 | GGGAGAGAGGTGGCTGTG | +140 : +157 |
| ASO-46 | GAAGAGGGAGAGAGGTGG | +145 : +162 |
| ASO-47 | TCAAGGAAGAGGGAGAGA | +150 : +167 |
| ASO-48 | AATGCTCAAGGAAGAGGG | +155 : +172 |
| ASO-49 | GACAAAATGCTCAAGGAA | +160 : +177 |
| ASO-50 | CATATGACAAAATGCTCA | +165 : +182 |
| ASO-51 | ATGACCATATGACAAAAT | +170 : +187 |
| **ASO-52** | **CACCAATGACCATATGAC** | **+175 : +192** |
| **ASO-53** | **TTAATCACCAATGACCAT** | **+180 : +197** |
| ASO-54 | TTTATTTAATCACCAATG | +185 : +202 |
| ASO-55 | TACATTTTATTTAATCAC | +190 : +207 |
| ASO-56 | TAAAATACATTTTATTTA | +195 : +212 |
| ASO-57 | AATATTAAAATACATTTT | +200 : +217 |
| ASO-58 | AAGTCAATATTAAAATAC | +205 : +222 |
| ASO-59 | AGAGAAAGTCAATATTAA | +210 : +227 |
| ASO-60 | GAAACAGAGAAAGTCAAT | +215 : +232 |
| ASO-61 | AGAAAGAAACAGAGAAAG | +220 : +237 |
| ASO-62 | AAGGTAGAAAGAAACAGA | +225 : +242 |
| ASO-63 | TTAAAAAGGTAGAAAGAA | +230 : +247 |
| ASO-64 | CATGTTTAAAAAGGTAGA | +235 : +252 |
| ASO-65 | GTAGCCATGTTTAAAAAG | +240 : +257 |
| ASO-66 | TTCTAGTAGCCATGTTTA | +245 : +262 |
| ASO-67 | CATTTTTCTAGTAGCCAT | +250 : +267 |

^ Efficient ASOs in bold.

*****Position relative to the *BIM* E3 5’-terminal nucleotide as +1, with positive and negative numbers respectively in exon and intron; the 3’ss polymorphic polyU tract is counted for a total of 14 Us as in the *BIM* minigenes.
